# Organoid technology demonstrates effects of potential drugs for COVID‐19 on the lung regeneration

**DOI:** 10.1111/cpr.12928

**Published:** 2020-10-19

**Authors:** Jianhai Wang, Xue Li, An Wang, Fuxiaonan Zhao, Qi Wu, Li Li, Hongzhi Yu, Junping Wu, Huaiyong Chen

**Affiliations:** ^1^ Department of Basic Medicine Haihe Hospital Tianjin University Tianjin China; ^2^ Department of Basic Medicine Haihe Clinical College of Tianjin Medical University Tianjin China; ^3^ Department of Tuberculosis Haihe Hospital Tianjin University Tianjin China; ^4^ Department of Respiratory Medicine Haihe Hospital Tianjin University Tianjin China; ^5^ Key Research Laboratory for Infectious Disease Prevention for State Administration of Traditional Chinese Medicine Tianjin Institute of Respiratory Diseases Tianjin China; ^6^ Tianjin Key Laboratory of Lung Regenerative Medicine Tianjin China

To the Editor,

Upon entry into the lungs, SARS‐CoV‐2 uses the angiotensin‐converting enzyme 2 (ACE2) receptor to facilitate viral entry into the epithelial cells that cover the airways and the alveolar gas‐exchanging space, leading to extensive epithelial injury, which contributes to local inflammatory storm and a series of respiratory syndromes.^1^ A number of drugs, based on their modes of action, have been suggested as therapeutic candidates for COVID‐19, and some of them are being evaluated in accelerated clinical trials.^1^ The antimalarial drugs chloroquine and hydroxychloroquine and the anti‐influenza drug umifenovir inhibit endocytosis of the virus. The HIV protease inhibitors lopinavir/ritonavir and broad‐spectrum antiviral agents ribavirin and favipiravir affect the ability of the virus to replicate in host cells. However, the efficacy and safety of these drugs are still controversial in the treatment of COVID‐19 patients.

The human lung harbours epithelial stem/progenitor cells that are activated to bring about the repair of airways and the respiratory epithelium after lung injury.^2^ Among them are airway club cells and alveolar type 2 (AT2) cells. The identity and distribution of club and AT2 cells are very similar in the lungs of mice and humans, thereby enabling successful evaluation of the functionality of human club and AT2 cells in mouse models.^3^ Club cells proliferate and differentiate into ciliated cells and goblet cells. At the terminal end of the respiratory tree, AT2 cells self‐renew and generate AT1 gas‐exchanging cells. Surprisingly, both club and AT2 cells are targeted by the SARS‐CoV‐2 because of abundant expression of ACE2 on the cell surface.^4^ Damage to the club and AT2 cells compromises the epithelial regenerative capacity in the lung. Insufficient epithelial repair could lead to progression of lung fibrosis, which is thought to occur in COVID‐19 patients.^5^ Stem/progenitor cell–derived three‐dimensional organoids serve not only as a model for evaluating stem/progenitor cell function in vitro, but also as a powerful platform for drug screening and safety assays. Much effort has been invested in the discovery of promising drug candidates and their testing in stringent clinical trials. However, care should be taken to select the right drug candidate that would not sacrifice the regenerative potential of the lung epithelium for better prognosis in COVID‐19 treatment.

Mouse club and AT2 cells were sorted and cultured in a 3D organoid‐based system as previously described (Figure 1A and B).^6^ Chloroquine was shown to inhibit the production of SARS‐CoV‐2 (EC_50_ = 1.13 μmol/L).^7^ Chloroquine did not affect colony‐forming efficiency (CFE) or the growth of organoids derived from club or AT2 cells (Figure 1C‐D and G‐H). The differentiation potential of club cells into ciliated cells (Foxj1) and goblet cells (Foxa3), and AT2 differentiation into AT1 cells (T1α) was also not affected (Figure 1E‐F and I).

**FIGURE 1:**
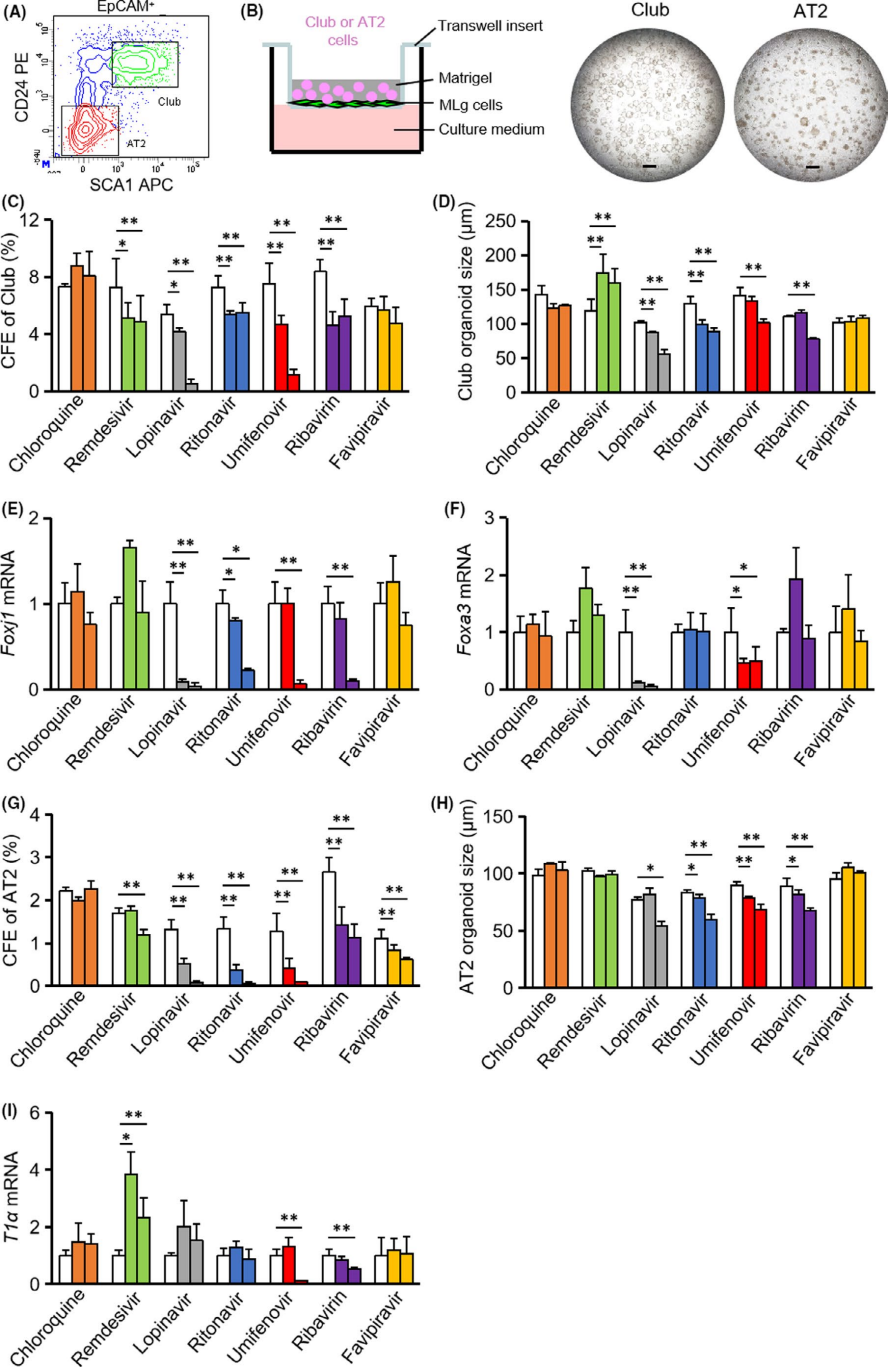
Drug candidates being tested against SARS‐CoV‐2 have various distinct effects on the viability, proliferation and differentiation of mouse progenitor cells. A, Club and AT2 cells were segregated from mouse lung epithelial cells by fluorescence‐activated cell sorting. B, Club cells and AT2 cells were cultured in a organoid platform, and organoids derived from club cells (day 7 after seeding) or from AT2 cells (day 10 after seeding) were imaged. C, G, Colony‐forming efficiency (CFE) of club cells or AT2 cells in the absence or presence of indicated potential drugs for COVID‐19 were calculated. D, H, Size of organoids derived from club cells or AT2 cells was quantified. E‐F, Foxj1 and Foxa3 mRNA expression was measured in club organoid cultures. I, T1α mRNA expression was measured in AT2 organoid cultures. Doses of drugs: chloroquine (0, 1, 10 μmol/L); remdesivir (0, 1, 10 μmol/L); lopinavir (0, 10, 50 μmol/L); ritonavir (0, 10, 50 μmol/L); umifenovir (0, 1, 10 μmol/L); ribavirin (0, 100, 500 μmol/L); and favipiravir (0, 50, 500 μmol/L). *P ˂ .05, **P ˂ .01; all data shown are means ± SD. Scale bar, 500 μm

The EC_50_ value of remdesivir (GS‐5734) against SARS‐CoV‐2 was estimated at 0.77 μmol/L.^7^ Remdesivir (at 1 and 10 μmol/L) decreased the CFE of club cells, but promoted the growth of club organoids with negligible effect on ciliated and goblet cell differentiation (Figure 1C‐F), the CFE of AT2 cells and the size of AT2 cell–derived organoids (Figure 1G‐H). Remdesivir enhanced AT1 cell differentiation at both doses, but reduced the CFE of AT2 cells at high doses (Figure 1G and I).

Lopinavir and ritonavir were confirmed to inhibit SARS‐CoV‐2 (EC_50_ values of lopinavir and ritonavir were 10.40 μmol/L and 8.63 μmol/L, respectively).^8^, ^9^ Lopinavir at 10 μmol/L decreased CFEs of both club and AT2 cells (Figure 1C and G). The growth of club cell‐ and AT2 cell–derived organoids was inhibited in the presence of lopinavir at 50 μΜ (Figure 1D and H). Lopinavir abrogated club cell differentiation but not AT2 cell differentiation (Figure 1E, F and I). Ritonavir reduced the CFE and organoid growth of both club and AT2 cells (Figure 1C‐D and G‐H). Ritonavir also suppressed ciliated cell differentiation, but goblet cell differentiation and AT1 differentiation remained unaffected (Figure 1E, F and I).

Similar to lopinavir, umifenovir (EC_50_ value against SARS‐CoV‐2 was 3.32 μmol/L) decreased the CFE and organoid growth of both club and AT2 cells in a dose‐dependent fashion (Figure 1C‐D and G‐H).^8^ Umifenovir at 10 μmol/L affected the differentiation potential of both club cells and AT2 cells was also not affected. Ribavirin was shown to inhibit SARS‐CoV‐2 with EC_50_ at 109.5 μmol/L.^7^ Ribavirin at the concentration of 100 μmol/L decreased CFEs of club and AT2 cells, and limited AT2 organoid growth (Figure 1C, G and H). Club organoid growth, ciliated cell differentiation and AT1 cell differentiation were affected by ribavirin at a higher dose (500 μmol/L) (Figure 1D‐F and I). Favipiravir exhibited antiviral activity against SARS‐CoV‐2 with an EC_50_ value of 61.88 μmol/L.^7^ Favipiravir (up to 500 μmol/L) had little effect on the proliferation and differentiation of club cells (Figure 1C‐F). Favipiravir inhibited the CFE of AT2 cells without affecting their organoid growth and AT1 differentiation (Figure 1G‐I).

Our overall data suggest that these drug candidates being tested against SARS‐CoV‐2 have various distinct effects on the viability, proliferation and differentiation of mouse progenitor cells that bring about epithelial regeneration in the lung. It will be interesting to further validate the effects of these drugs in organoid cultures with human club and AT2 cells. The clinical efficacy of these drugs as established by current trials is still controversial and needs to be further optimized in terms of dosage, timing of administration and administration routes.^10^ Considering that primary human lung progenitor cells are usually hard to access, mouse organoid systems used in this study lay the foundation for further evaluation of existing and experimental drugs, in order to achieve better prognosis against SARS‐CoV‐2 infections. Future optimization of this organoid technology, by using human ACE2 transgenic mouse or establishing organoid infection model with virus expressing human ACE2, will definitely accelerate its application in the war against the pandemic.

## CONFLICT OF INTEREST

The authors have no financial conflicts of interest.

## AUTHORS’ CONTRIBUTIONS

XL, JWang, HY, JWu and HC designed the experiments and analysed the data. JWang, XL, AW and FZ performed the experiments. HC and JW wrote the manuscript. HY, JWu and LL revised the manuscript.

### DATA AVAILABILITY STATEMENT

The authors declare that all the data supporting the findings of this study are available within the article and its Supplementary Information files and from the corresponding authors on reasonable request.
